# *TERT* Promoter Mutations Are an Independent Predictor of Distant Metastasis in Middle Eastern Papillary Thyroid Microcarcinoma

**DOI:** 10.3389/fendo.2022.808298

**Published:** 2022-03-11

**Authors:** Sandeep Kumar Parvathareddy, Abdul K. Siraj, Kaleem Iqbal, Zeeshan Qadri, Saeeda O. Ahmed, Maha Al-Rasheed, Ahmed A. AlQatie, Saif S. Al-Sobhi, Fouad Al-Dayel, Khawla S. Al-Kuraya

**Affiliations:** ^1^ Human Cancer Genomic Research, Research Center, King Faisal Specialist Hospital and Research Center, Riyadh, Saudi Arabia; ^2^ Department of Radiology, King Faisal Specialist Hospital and Research Center, Riyadh, Saudi Arabia; ^3^ Department of Surgery, King Faisal Specialist Hospital and Research Center, Riyadh, Saudi Arabia; ^4^ Department of Pathology, King Faisal Specialist Hospital and Research Centre, Riyadh, Saudi Arabia

**Keywords:** papillary thyroid microcarcinoma, *TERT* promoter mutations, distant metastasis, metastasis-free survival, predictor

## Abstract

**Background:**

Papillary thyroid microcarcinomas (PTMCs) have been attributed to the recent increased incidence of thyroid cancer. Although indolent, a subset of PTMC could potentially develop distant metastasis (DM). This study aimed to evaluate the clinico-pathological features and molecular characteristics of PTMC and identify the risk factors for DM in PTMC patients from Middle Eastern ethnicity.

**Methods:**

We retrospectively analyzed 210 patients with histologically confirmed PTMC. Clinico-pathological associations for DM, *BRAF* mutation and *TERT* mutation were analyzed successfully in 184 patients. Multivariate analysis was performed using Cox proportional hazards model and logistic regression analysis.

**Results:**

Among the PTMC patients included in this cohort, DM was noted in 6.0% (11/184), whereas tumor relapse occurred in 29/184 (15.8%). Of the 11 cases with DM, lung metastasis occurred in 8 cases, bone metastasis in 2 cases and brain metastasis in 1 case. Presence of extrathyroidal extension and male sex were significantly associated with DM. Molecular analysis showed *BRAF V600E* mutations to be the most frequent, being detected in 45.7% (84/184). *TERT* promoter mutations were detected in 16 (8.7%) cases and were significantly associated with DM and shorter metastasis-free survival in multivariate analysis.

**Conclusions:**

Our study indicates a surprisingly high frequency of *TERT* promoter mutation in Saudi patients with PTMC. Identifying *TERT* promoter mutations as an independent predictor of DM in patients with microcarcinoma could explain the inherent aggressive nature of PTMC from Middle Eastern ethnicity and magnify its role in patient risk stratification, which might help in improving therapeutic strategy for these patients.

## Introduction

Papillary Thyroid Carcinoma (PTC) is the commonest subtype of thyroid cancer (1). The incidence of PTC has increased over the past decades (2–4). Papillary thyroid microcarcinoma (PTMC) is defined as PTC measuring ≤ 1cm along the largest diameter (5). Important advancements in diagnostic imaging and increased thoroughness of pathological examination have led to increased prevalence of PTMC among newly diagnosed PTC (6–8). Despite the increasing incidence of PTMC, it has an indolent clinical course, with good prognosis and favorable patient outcome (4, 9–11). Given the indolent nature of PTMC, it is anticipated the majority of PTMC will not cause clinically significant disease during patients’ lifetime and distant metastasis (DM) from PTMC has been considered a rare occurrence (10, 12, 13).

Several clinico-pathological features such as extrathyroidal extension, multifocality, lymph node metastasis, vascular invasion, and aggressive histological subtypes have been proposed as prognostic factors for PTMC recurrence (14–17). However, incidence and risk factors for distant metastasis remain to be further illustrated, especially in ethnicities where thyroid cancer is very common, such as the Middle Eastern ethnicity. In fact, PTC is very common in Saudi Arabia and ranks as the second most common cancer affecting females, after breast cancer (18). Therefore, identifying the incidence and risk factors for PTMC DM in Middle Eastern ethnicity is urgently needed. Indeed, it is noteworthy that PTC from Middle eastern ethnicity is unique with regards to being inherently more aggressive, as evidenced by other studies from this region, which also found a relatively high rate of aggressive variants (19, 20), multifocality (21, 22), extrathyroidal extension (19, 23), distant metastasis (19, 23) and a low median age at diagnosis (24, 25). A study by Gul et al. (26) in a Middle Eastern (Turkish) population assessed the difference in clinico-pathological characteristics between PTMC and PTC. They found that the incidence of capsular invasion, vascular invasion, extrathyroidal extension, lymph node involvement and multifocality was significantly lower in PTMC compared to PTC.

In this retrospective study, we evaluated the various clinico-pathological features to discriminate which PTMC will have more aggressive behavior. We then deepen the analysis of those PTMC with DM, focusing on identifying molecular markers for DM from PTMC. It is known that the initiation of PTC involves primary driver mutations, of which the most common are *mitogen-activated protein (MAP) kinase* mutations (*BRAF V600E* and *RAS* mutations) (27, 28). However, the development of more aggressive PTC is suggested to be associated with additional late driver mutations like telomerase reverse transcriptase (*TERT*) promoter mutations (29, 30). Previous studies have evaluated the mutational profile of PTMC with aggressive features with contrasting results (31–33). *BRAF* is the most studied gene in PTMC, being detected in about 50% of cases and was associated with aggressive clinical features and risk of recurrence (32, 34, 35). However, incidence of *TERT* promoter mutations and their clinical impact on PTMC have shown contradicting results. While the presence of *TERT* promoter mutations have been reported at a low rate in some studies (36, 37), others failed to find any *TERT* mutations in PTMC (38). The conflicting results about the clinical impact of *TERT* mutations in PTMC also exist. A previous report has shown close correlation between *TERT* mutations and unfavorable outcomes such as recurrence and DM (37), whereas others could not identify the correlation between *TERT* promoter mutations and PTMC patients’ outcome (36, 39).

Therefore, we have conducted this study to analyze the clinico-pathological and molecular characteristics of Middle Eastern PTMC patients. The aim of this study was to report the clinical outcomes in PTMC and to identify factors that can be predictive of distance metastasis in these patients. If predictive markers could be identified at presentation using pre-surgical fine-needle aspiration specimens, the management of PTMC patients will improve, thereby leading to better surgical and therapeutic interventions.

## Materials and Methods

### Patient Selection

Two hundred and ten PTMC patients diagnosed between 1988 and 2018 at King Faisal Specialist Hospital and Research Centre (Riyadh, Saudi Arabia) were available to be included in the study. However, molecular data (*TERT*, *RAS* and *BRAF* mutation) were successfully analyzed in 184 cases and hence were included in the study. Cases were identified based on clinical history followed by fine needle aspiration cytology for confirmation. The Institutional Review Board of the hospital approved this study and the Research Advisory Council (RAC) provided waiver of consent under project RAC # 2211168 and # 2110031.

### Clinico-Pathological Data

Baseline clinico-pathological data were collected from case records and have been summarized in **Table 1**. Staging of PTC was performed using the eighth edition of American Joint Committee on Cancer (AJCC) staging system. Based on the 2015 American Thyroid Association (ATA) guidelines, tall cell, hobnail, columnar cell, diffuse sclerosing and insular variants were classified as aggressive variants, whereas classical and follicular variants were classified as non-aggressive variants (40), for multivariate analysis. DM was either synchronous (at the time of diagnosis) or metachronous (developed during follow-up). Of the 11 cases showing DM, four cases were diagnosed by computed tomography (CT) scan and further confirmed by histopathological examination, whereas the remaining seven cases were diagnosed by 131-I whole body scan, which showed iodine absorption in distant metastatic lesions. Only structural recurrence (local, regional or distant) was considered for analysis. Recurrence was defined as any newly detected tumor (local or distant) or metastatic regional lymph node (LN), based on ultrasound and/or imaging studies in patients who had been previously free of disease following initial treatment. Risk stratification was done based on 2015 ATA guidelines, as low-, intermediate- and high-risk PTC.

**Table 1:** Clinico-pathological and molecular characteristics of Papillary thyroid microcarcinoma.

	No.	%
**Total**	184	
**Age (years)**		
Median (range)	40.1 (12 – 84)	
<55	154	83.7
≥55	30	16.3
**Gender**		
Female	147	79.9
Male	37	20.1
**Histologic subtype**		
Classical variant	130	70.7
Follicular variant	31	16.8
Tall cell variant	16	8.7
Other variants	7	3.8
**Tumor laterality**		
Unilateral	121	65.8
Bilateral	63	34.2
**Multifocality**		
No	111	60.3
Yes	73	39.7
**Extrathyroidal extension**		
Present	63	34.2
Absent	121	65.8
**Lymphovascular invasion**		
Present	26	14.1
Absent	158	85.9
**Tumor size (cm)**		
≤0.5	43	23.4
>0.5	141	76.6
**Lymph node metastasis**		
N0	79	42.9
N1	85	46.2
Nx	20	10.9
**Distant metastasis**		
Absent	173	94.0
Present	11	6.0
Synchronous	5	2.7
Metachronous	6	3.3
**Site of Distant metastasis**		
Lung	8	72.7
Bone	2	18.2
Brain	1	9.1
**TNM Stage**		
I	165	90.7
II	11	6.0
III	2	1.1
IV	4	2.2
***BRAF* mutation**		
Present	84	45.7
Absent	100	54.3
***NRAS* mutation**		
Present	14	7.6
Absent	170	92.4
***HRAS* mutation**		
Present	2	1.1
Absent	182	98.9
***KRAS* mutation**		
Present	2	1.1
Absent	182	98.9
***TERT* mutation**		
Present	16	8.7
Absent	168	91.3
**Recurrence**		
Yes	29	15.8
No	155	84.2
**ATA risk category**		
Low risk	46	25.0
Intermediate risk	77	41.8
High risk	61	33.2
**Type of surgery**		
Thyroidectomy only	20	10.9
Thyroidectomy + ND	164	89.1
**RAI given**		
Yes	139	75.5
No	45	24.5
**Total follow-up duration**		
Median (range) (in years)	9.3 (1.0 – 28.6)	

ATA, American Thyroid Association; RAI, Radioactive iodine.

### DNA Isolation

DNA was isolated from formalin-fixed, paraffin-embedded (FFPE) tumor tissues using Gentra DNA isolation kit (Gentra, Minneapolis, MN, USA), following the manufacturer’s recommendations.

### PCR and Sanger Sequencing

Primer 3 software was used to design the primers for the two hotspot mutations (C228T and C250T) in promoter region of *TERT* gene along with entire coding and splicing regions of *MAPK* genes exons 2 and 3 in *HRAS*, *KRAS* and *NRAS* and exon 15 in *BRAF* (**Supplementary Table 1**). PCR was performed in a total volume of 25 µl with 20 ng of genomic DNA, 2.5 µl 10 x Taq buffer, 2.3 mM dNTPs, 1 unit Taq polymerase and 0.2 µM each primer and de-ionized water. The efficiency and quality of the amplified PCR products was confirmed by loading them on a 2% agarose gel.

The PCR products were subsequently subjected to direct sequencing with BigDye terminator V 3.1 cycle sequencing reagents and analyzed on an ABI 3730XL DNA analyzer (Applied Biosystems, Foster City, CA). Reference sequences were downloaded from NCBI GenBank. Sequencing traces were analyzed with the Mutation Surveyor v4.04 (Soft Genetics, LLC, State College, PA).

### Follow-Up and Study Endpoint

Patients were regularly followed-up by both physical examinations and imaging studies to identify tumor recurrence. The median follow-up was 9.3 years (range 1.0 – 28.6 years). Metastasis-free survival (MFS) was defined as the time (in months) from date of initial surgery to the occurrence of any DM. In case of no DM, date of last follow-up was the study endpoint for MFS.

### Statistical Analysis

The associations between clinico-pathological variables were performed using contingency table analysis and Chi square tests. Mantel-Cox log-rank test was used to evaluate MFS. Survival curves were generated using the Kaplan-Meier method. Cox proportional hazards model (extended and restricted) and logistic regression was used for multivariate analysis. Since stage is a composite variable (consisting of age, tumor size, extrathyroidal extension, LN metastasis and DM), we performed multivariate Cox proportional hazards by dividing it into two models. The extended model included age, histology, tumor focality, extrathyroidal extension, lymphovascular invasion, pT, lymph node metastasis, *BRAF* mutation and *TERT* mutation as co-variates, whereas the restricted model consisted of histology, tumor focality, lymphovascular invasion, stage, *BRAF* mutation and *TERT* mutation as co-variates. Backward elimination was applied to select variables for inclusion in the multivariate Cox proportional hazards model. Two-sided tests were used for statistical analyses with a limit of significance defined as p value < 0.05. Data analyses were performed using the JMP14.0 (SAS Institute, Inc., Cary, NC) software package.

## Results

### Patient and Tumor Characteristics

**Figure 1:**
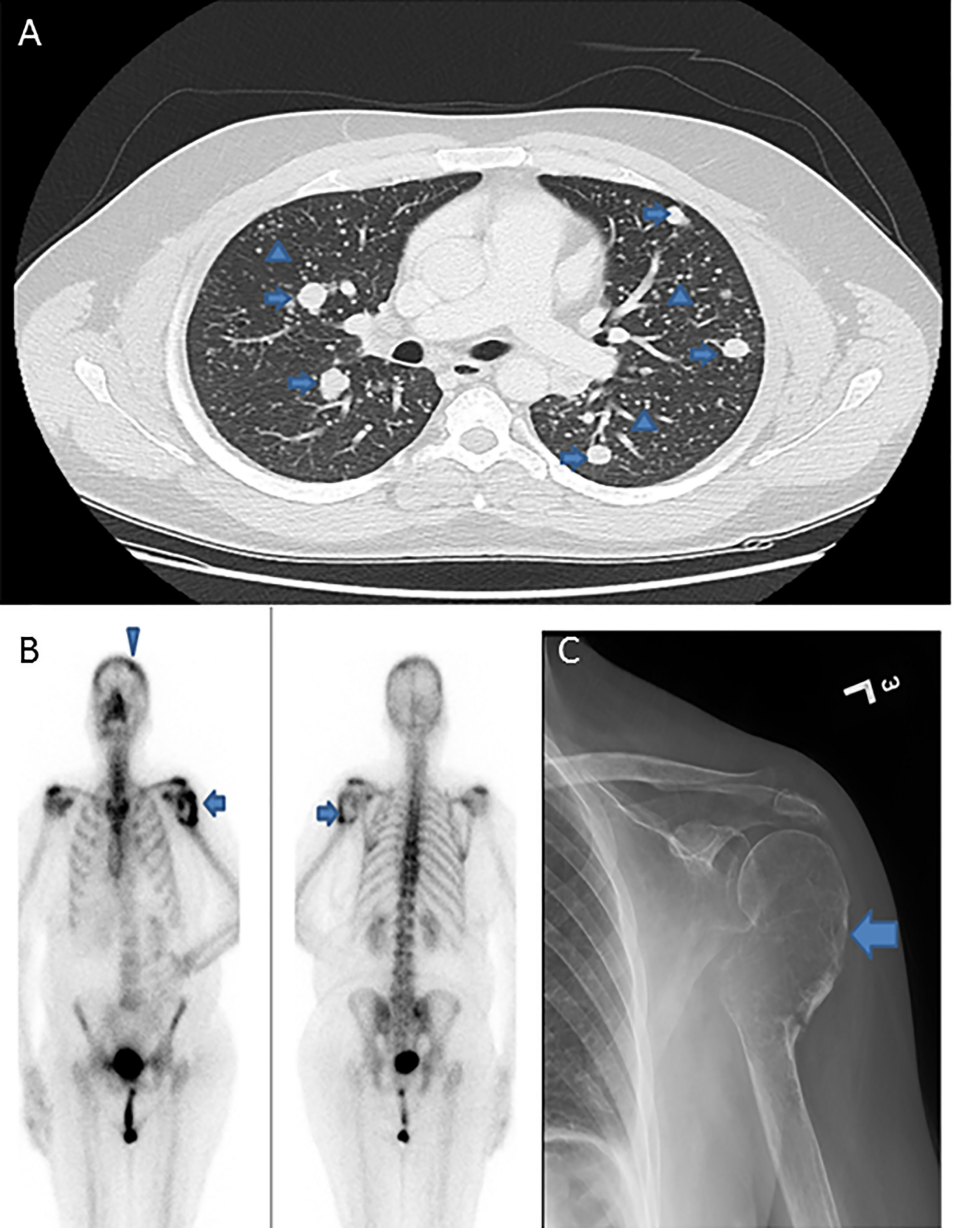
Radiologic images of distant metastasis in papillary thyroid microcarcinoma cases. **(A)** Computed tomography of the lung showing extensive pulmonary metastases with classic canon-ball metastases (arrows) and miliary metastatic nodules (arrow heads). **(B)** Positron emission tomography (PET) scan and **(C)** X-ray, showing expansile lytic metastatic lesion in the left humerus (arrows) with pathologic fracture related to metastasis. PET scan also shows a metastatic focus in the skull bone (arrow head).

Median age of the study population was 40.1 years (range: 12 – 84 years), with a male to female ratio of 1:4. The majority of tumors were classical variant of PTC (70.7%; 130/184). 34.2% (63/184) of tumors were bilateral and 39.7% (73/184) were multifocal. 34.2% (63/184) of tumors exhibited extrathyroidal extension and 14.1% (26/184) showed lymphovascular invasion. Tumor recurrence was seen in 15.8% (29/184). DM was noted in 6.0% (11/184), with 2.7% (5/184) of them being synchronous and 3.3% (6/184) being metachronous. DM to the lung was most common, accounting for 72.7% (8/11), followed by bone (18.2%; 2/11) and brain (9.1%; 1/11) (**Figure 1** and **Table 1**).

### Clinico-Pathological Associations of Distant Metastasis in PTMC

DM was noted in 6.0% (11/184) of PTMCs and was significantly associated with male sex (p = 0.0088) and extrathyroidal extension (p = 0.0008). On further analysis with molecular markers, we found that DM in PTMC was associated with *TERT* mutation (p < 0.0001) but not *MAP kinase* gene mutations (*BRAF*, p = 0.1969; *NRAS*, p = 0.8522; *HRAS*, p = 0.0801; KRAS, p = 1.0000) (**Table 2**).

**Table 2:** Clinico-pathological associations of distant metastasis in papillary thyroid microcarcinoma.

			Distant metastasis				p value
	Total		Present		Absent		
	No.	%	No.	%	No.	%	
**Total**	184		11	6.0	173	94.0	
**Age at surgery (years)**							
<55	154	83.7	7	63.6	147	85.0	0.0947
≥55	30	16.3	4	36.4	26	15.0	
**Gender**							
Male	37	20.1	6	54.5	31	17.9	0.0088
Female	147	79.9	5	45.5	142	82.1	
**Histologic subtype**							
Classical variant	130	70.6	7	63.6	123	71.1	0.6613
Follicular variant	31	16.9	3	27.3	28	16.2	
Tall cell variant	16	8.7	1	9.1	15	8.7	
Other variants	7	3.8	0	0.0	7	4.0	
**Extrathyroidal extension**							
Present	63	34.2	9	81.8	54	31.2	0.0008
Absent	121	65.8	2	18.2	119	68.8	
**Lymphovascular invasion**							
Present	26	14.1	2	18.2	24	13.9	0.7005
Absent	158	85.9	9	81.8	149	86.1	
**Tumor focality**							
Unifocal	111	60.3	7	63.6	104	60.1	0.8161
Multifocal	73	39.7	4	36.4	69	39.9	
**Tumor laterality**							
Unilateral	121	65.8	5	45.5	116	67.0	0.1549
Bilateral	63	34.2	6	54.5	57	33.0	
**Lymph node metastasis**							
Present	85	51.8	8	72.7	77	50.3	0.1430
Absent	79	48.2	3	27.3	76	49.7	
**TNM Stage**							
I	165	90.7	6	60.0	159	92.4	0.0021
II	11	6.0	1	10.0	10	5.8	
III	2	1.1	0	0.0	2	1.2	
IV	4	2.2	3	30.0	1	0.6	
***TERT* mutation**							
Present	16	8.7	6	54.5	10	5.8	< 0.0001
Absent	168	91.3	5	45.5	163	94.2	
***BRAF* mutation**							
Present	84	45.7	3	27.3	81	46.8	0.1969
Absent	100	54.3	8	72.7	92	53.2	
***NRAS* mutation**							
Present	14	7.6	1	9.1	13	7.5	0.8522
Absent	170	92.4	10	90.9	160	92.5	
***HRAS* mutation**							
Present	2	1.1	1	9.1	1	0.6	0.0801
Absent	182	98.9	10	90.9	172	99.4	
***KRAS* mutation**							
Present	2	1.1	0	0.0	2	1.2	1.0000
Absent	182	98.9	11	100.0	171	98.8	

### Clinico-Pathological Associations of *BRAF* and *TERT* Mutations in PTMC

We next analyzed the clinico-pathological associations of *BRAF* and *TERT* mutations in our cohort. *BRAF* mutation was seen in 45.7% (84/184) of PTMCs. *BRAF* mutations were significantly associated with tall cell variant of PTC (P = 0.0140), extrathyroidal extension (p = 0.0014), multifocality (p = 0.0004), bilaterality (p = 0.0039) and presence of lymph node metastasis (p = 0.0067). However, no association was seen with DM (p = 0.1969) or tumor recurrence (p = 0.4753) (**Supplementary Table 2**).

*TERT* promoter mutations were seen in 8.7% (16/184) of PTMCs and were significantly associated with adverse clinico-pathological characteristics, such as older age (p = 0.0001), extrathyroidal extension (p = 0.0156), DM (p < 0.0001), stage IV tumors (p < 0.0001) and tumor recurrence (p = 0.0048) (**Table 3**).

**Table 3:** Clinico-pathological associations of *TERT* mutation in papillary thyroid microcarcinoma.

			*TERT* mutation				p value
	Total		Present		Absent		
	No.	%	No.	%	No.	%	
**Total**	184		16	8.7	168	91.3	
**Age at surgery (years)**							
<55	154	83.7	7	43.8	147	87.5	0.0001
≥55	30	16.3	9	56.2	21	12.5	
**Gender**							
Male	37	20.1	5	31.3	32	19.1	0.2686
Female	147	79.9	11	68.7	136	80.9	
**Histologic subtype**							
Classical variant	130	70.6	10	62.5	120	71.4	0.3828
Follicular variant	31	16.9	3	18.8	28	16.7	
Tall cell variant	16	8.7	3	18.8	13	7.7	
Other variants	7	3.8	0	0.0	7	4.2	
**Extrathyroidal extension**							
Present	63	34.2	10	62.5	53	31.6	0.0156
Absent	121	65.8	6	37.5	115	68.4	
**Lymphovascular invasion**							
Present	26	14.1	3	18.8	23	13.7	0.5925
Absent	158	85.9	13	81.2	145	86.3	
**Tumor focality**							
Unifocal	111	60.3	7	43.8	104	61.9	0.1613
Multifocal	73	39.7	9	56.2	64	38.1	
**Tumor laterality**							
Unilateral	121	65.8	7	43.8	114	67.9	0.0591
Bilateral	63	34.2	9	56.2	54	32.1	
**Lymph node metastasis**							
Present	85	51.8	8	53.3	77	51.7	0.9026
Absent	79	48.2	7	46.7	72	48.3	
**Distant metastasis**							
Present	11	6.0	6	37.5	5	3.0	< 0.0001
Absent	173	94.0	10	62.5	163	97.0	
**TNM Stage**							
I	165	90.7	10	62.5	155	93.4	< 0.0001
II	11	6.0	2	12.5	9	5.4	
III	2	1.1	0	0.0	2	1.2	
IV	4	2.2	4	25.0	0	0.0	
***BRAF* mutation**							
Present	84	45.7	10	62.5	74	44.1	0.1569
Absent	100	54.3	6	37.5	94	55.9	
**Recurrence**							
Yes	29	15.8	7	43.8	22	13.1	0.0048
No	155	84.2	9	56.2	146	86.9	

### *TERT* Promoter Mutations Are Independent Predictors for Distant Metastasis and Metastasis-Free Survival in PTMC

**Figure 2:**
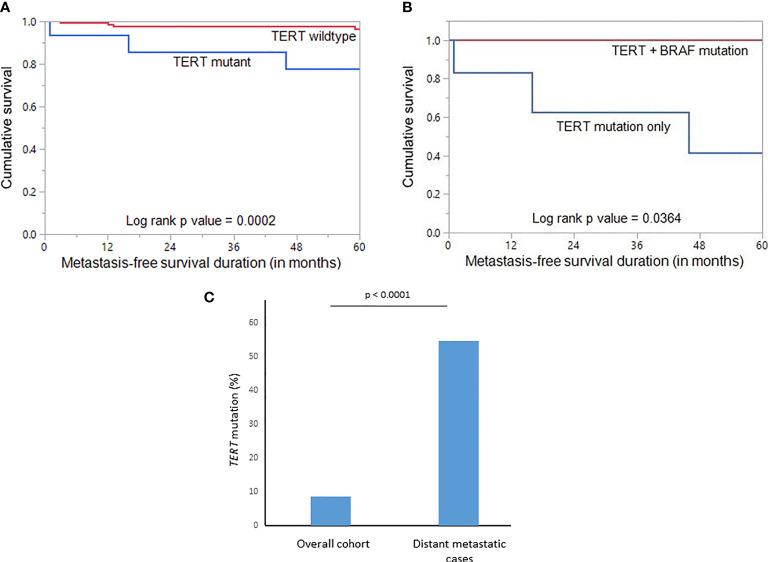
**(A)** Kaplan-Meier survival curve showing shorter metastasis-free survival in TERT mutant cases compared to TERT wildtype (p = 0.0002). **(B)** Kaplan-Meier survival curve showing shorter metastasis-free survival in TERT mutant only cases compared to TERT+BRAF mutant cases (p = 0.0364). **(C)** Significantly higher frequency of TERT mutation was noted in the distant metastatic cases compared to the overall cohort of papillary thyroid microcarcinomas (p < 0.0001).

Given the fact that the frequency of both DM and *TERT* promoter mutations was relatively high in our cohort and also were significantly associated with each other, we sought to further analyze the relationship between them. Using Mantel Cox log rank test, we found that *TERT* mutations were associated with shorter metastasis-free survival (p = 0.0002; **Figure 2A**). Next, we sought to analyze whether this prognostic effect was due to co-existing *BRAF* mutation. In our PTMC series, coexistence of *BRAF* and *TERT* promoter mutations was found in 5.4% (10/184) of the cases. We found that patients with *TERT* mutation alone had a significantly shorter metastasis-free survival compared to patients with *TERT+BRAF* mutations (p = 0.0364; **Figure 2B**), suggesting that *TERT* mutations had a significantly greater impact on clinical outcome in PTMC.

In multivariate Cox proportional hazards analysis using the extended model, LN metastasis (p = 0.0076) and *TERT* mutation (p = 0.0249) were found to be significant, whereas in multivariate analysis of the restricted model, only *TERT* mutation was an independent predictor for metastasis-free survival (Hazard ratio = 20.88, 95% confidence interval = 3.43 – 146.49, p = 0.0015) (**Table 4**).

**Table 4:** Multivariate analysis using Cox proportional hazards model for metastasis-free survival.

Clinico-pathological variables	Multivariate (extended)		Multivariate (restricted)	
	HR(95% CI)	p value	HR(95% CI)	p value
**Age**				
≥ 55 years (*vs*. < 55 years)	5.62 (0.32-153.53)	0.2282		
**Histology**				
Tall cell variant (*vs*. other variants)	0.06 (0.01-1.55)	0.0948	0.20 (0.01-3.49)	0.3070
**Tumor focality**				
Multifocal (*vs*. Unifocal)	0.13 (0.01-0.91	0.0402	0.73 (0.13-3.29)	0.6884
**Extrathyroidal extension**				
Present (*vs*. Absent)	3.58 (0.23-50.18	0.3415		
**Lymphovascular invasion**				
Present (*vs*. Absent)	5.85 (0.22-90.93)	0.2442	5.88 (0.26-62.90)	0.2157
**pT**				
T3/4 (*vs*. T1)	7.29 (0.86-95.64)	0.0692		
**Lymph node metastasis**				
Present (*vs*. absent)	14.98 (1.87-469.34)	0.0076		
**Stage**				
III-IV (*vs*. I-II)			28.49 (2.26-340.55)	0.0121
***BRAF* mutation**				
Present (*vs*. Absent)	0.10 (0.01-1.11)	0.0622	0.17 (0.01-1.17)	0.0739
***TERT* mutation**				
Present (*vs*. Absent)	11.99 (1.39-145.21)	0.0249	20.88 (3.43-146.49)	0.0015

HR, Hazard ratio; CI, Confidence interval.

Using multivariate logistic regression analysis, *TERT* mutation was also found to be an independent predictor for DM (Odds ratio = 138.87, 95% confidence interval = 5.02 – 3839.70, p = 0.0036) (**Table 5**). To further support the predictive power of *TERT* mutation for DM, we analyzed the frequency of *TERT* mutations in the overall cohort and distant metastatic cases alone. We found that the frequency of *TERT* mutations in the distant metastatic cases was more than 6-fold higher compared to the overall cohort (54.5% *vs*. 8.7%, p < 0.0001; **Figure 2C**).

**Table 5:** Multivariate analysis using logistic regression model for prediction of distant metastasis.

Clinico-pathological variables	Distant metastasis		
	Odds Ratio	95% Confidence interval	p value
**Age**			
≥ 55 years (*vs*. < 55 years)	0.86	0.02 – 33.05	0.9358
**Histology**			
Tall cell variant (*vs*. other variants)	0.04	0.00 – 4.46	0.1764
**Tumor focality**			
Multifocal (*vs*. Unifocal)	0.14	0.02 – 1.21	0.0742
**Extrathyroidal extension**			
Present (*vs*. Absent)	32.81	1.11 – 967.46	0.0432
**Lymphovascular invasion**			
Present (*vs*. Absent)	1.13	0.88 – 14.63	0.9231
**pT**			
T3/4 (*vs*. T1)	2.95	0.26 – 33.65	0.3835
**Lymph node metastasis**			
Present (*vs*. absent)	5.44	0.41 – 72.52	0.1997
**Stage**			
III-IV (*vs*. I-II)	6.17	0.05 – 713.89	0.4527
***BRAF* mutation**			
Present (*vs*. Absent)	19.29	0.90 – 413.38	0.0584
***TERT* mutation**			
Present (*vs*. Absent)	138.87	5.02 – 3839.70	0.0036

## Discussion

PTMC has shown a sharp rise in incidence in recent decades (2–4, 41). Current therapeutic approaches range from active surveillance to surgery and RAI therapy depending on risk stratification of PTC tumors (42–44). Although PTMC is frequently reported to have an excellent outcome (9–11), identifying aggressive PTMC is still challenging. Unfortunately, identifying a way to accurately predict the outcome of PTMC patients is still debatable and has not reached a consensus. We have conducted this current study to identify the incidence, clinical, pathological, and molecular features of Middle Eastern PTMC and detect if any of these characteristics could help to predict tumor behavior.

We analyzed 184 Middle Eastern patients affected by PTMC who were followed for a median duration of 9.2 years. All patients had undergone thyroidectomy, with 75.5% (139/184) of patients receiving RAI therapy. Although we observed aggressive clinical features in 63.6% of the PTMCs, our cohort had a favorable prognosis as a whole (5-year cancer-specific survival was 100% and 5-year disease-free survival was 80.3%). Nevertheless, persistent disease was found in 12.1% of PTMC patients and recurrence was observed in 15.8%, mostly loco-regional, with a mean duration of 4.2 years. This relatively high incidence of recurrence could be explained by the fact that nearly one-third of patients presented with ATA high risk disease, which is not seen in most modern studies from Western population and may be partially attributed to genetics or differences in presentation and access to health care.

Interestingly, incidence of DM in this cohort was 6% among PTMC patients (3.3% patients developed DM after a median of 3.2 years of initial diagnosis while 2.7% presented with DM at diagnosis). This relatively high incidence rate among Middle Eastern PTMC patients is in contrast to previous reports from other ethnicities, where DM from PTMC was very rare (10, 12, 45–48). We were intrigued by the occurrence of distance metastasis at this relatively high rate. Therefore, we further characterized the clinico-pathological and molecular factors that are associated with DM in PTMC. We identified male sex and extrathyroidal extension as risk factors for DM in PTMC. The majority of DM (72.7%; 8/11) were seen in the lungs. This is of clinical importance since it suggests that PTMC should be considered in the differential diagnosis for primary cancer when metastatic lesions from an unknown origin are seen.

Furthermore, genetic profile analysis of PTMCs showed that the most common mutations were *MAP kinase* pathway gene alterations (*BRAF* mutation in 45.7%, followed by *NRAS* mutation in 7.6%). *BRAF* mutation in PTC from the Middle East has been reported to range from 40% - 71% (49–51), with the corresponding frequency in PTMC ranging from 18% - 72% (52–54) in published literature from this region, which is in concordance with our findings. Our study demonstrated a significant correlation between *BRAF* mutations and aggressive histopathological features such as tall cell variant, bilaterality, multifocality, extrathyroidal extension and LN metastasis. However, no association with DM or clinical outcome was found in this cohort. Previous studies have rarely analyzed genes other than *MAP kinase* pathway genes (31, 33). Several studies have reported that mutations in *TERT* promoter are associated with aggressive clinico-pathological features in PTC, especially if they co-exist with *BRAF* mutations (55–57). However, the significance of *TERT* promoter mutations in predicting aggressiveness of PTMC has not been fully illustrated and requires further investigation.

In the present study, mutations of *TERT* promoter were successfully analyzed in 184 PTMCs and the prevalence was 8.7%. This finding is surprising, considering the fact that *TERT* mutations are late events in thyroid carcinogenesis and their presence is usually seen in large and more advanced PTCs. The prevalence of *TERT* promoter mutations was relatively high compared to previous studies in the literature from PTMC patients of different ethnic backgrounds. De Biase et al. (36) have reported the prevalence of *TERT* promoter mutations in 4.7% (19/404) PTMC from Italy, whereas Sama et al. (45) found the prevalence to be 2% (2/100) in PTMC from the same population. Three other studies from Asian population reported a frequency of 3.2% (16/504) from Korea (39), 1.2% (1/86) from China (37) and 0% (0/26) from Japan (38). We have previously reported a frequency of 18.0% for *TERT* promoter mutations in a large cohort of PTCs (n = 927) (55). However, data on incidence of *TERT* mutations in PTMC from Middle eastern ethnicity is lacking. These differences in the mutation frequency could be attributed to various factors, including cohort size, environmental factors as well as geographical and ethnic variations.

None of the above studies have shown significant association between *TERT* promoter mutation and aggressive clinico-pathological characteristics in PTMC. In contrast, the present study showed *TERT* promoter mutations were significantly associated with aggressive clinico-pathological features, such as older age, extrathyroidal extension, tumor recurrence and DM. In fact, we demonstrated using Cox regression analysis that *TERT* promoter mutations were an independent prognostic marker for MFS. *TERT* mutations were significant in both the extended and restricted models, which confirms the prognostic value of *TERT* promoter mutations as an independent marker for MFS. In addition, we also found that *TERT* promoter mutations were an independent predictive marker for DM in PTMC using logistic regression analysis. Supporting this novel finding in this ethnicity, we further analyzed the frequency of *TERT* promoter mutations in distant metastatic PTMC. Upon comparing the frequency of *TERT* promoter mutations in the original unselected cohort and PTMC with documented DM, we found *TERT* promoter mutations frequency increased by more than 6-fold in the latter group (54.5% *vs*. 8.7% p < 0.0001). Similar findings have been reported in PTC by Melo et al. (58) and others (37, 55, 59), where they show that *TERT* promoter mutations are a marker for DM. Studies by Povoa et al. (60) and Melo et al. (61) analyzed the association of *TERT* and *BRAF* mutations with distant metastasis in PTC. They found that while *TERT* mutations were associated with distant metastasis, *BRAF* mutations were not, which is in concordance with our findings. We also found that patients with *TERT* mutation alone had a significantly shorter metastasis-free survival compared to patients with coexisting *TERT* and *BRAF* mutations. Taken together, these data suggest that presence of *TERT* promoter mutations in PTMC might reflect true aggressive biological subtype of PTC in this ethnicity. Additional larger studies are required to confirm this assumption.

Despite the limitations of this study being a retrospective single institute study, and the relatively small number of cases, it has several strengths, including the unique PTMC cohort from Middle Eastern ethnicity, long follow-up time and comprehensive follow-up information on recurrence as well as detailed histopathological and molecular information. However, owing to the aggressive behavior of PTMCs in our cohort, the finding may not be applicable to general PTMCs. Further confirmation with a larger sample size is encouraged.

In conclusion, patients with PTMC from this ethnicity harbor aggressive features and may develop distance metastasis, in particular lung metastases. Screening for distant metastasis should be considered, especially in patients harboring *TERT* promoter mutations. The available evidence indicates *TERT* promoter mutations may have great value in improving the risk stratification and predication DM and MFS of patients with aggressive PTMC. Therefore, mutation in the *TERT* promoter may be an important factor in the genetic background, driving aggressiveness of PTMC from this ethnicity and detection of such mutations may help in the accurate identification and management of PTMC in this population.

## Data Availability Statement

The original contributions presented in the study are included in the article/**Supplementary Material**. Further inquiries can be directed to the corresponding author.

## Ethics Statement

The studies involving human participants were reviewed and approved by Research Advisory Council, King Faisal Specialist Hospital and Research Centre. Written informed consent for participation was not provided by the participants’ legal guardians/next of kin because: Since only retrospective clinical data was utilized and patients were de-identified, a waiver of written consent was granted for this study.

## Author Contributions

Study concept and design: KA-K, SP, and AS. Executed the study: SP, AS, KI, SA, MA-R, AA, SA-S, and FA-D. Statistical analysis: ZQ. Drafting the article: KA-K, SA, AS, and SP. Critical revision of the article for important intellectual content, writing of the article, and approval of the final version: KA-K, SP, AS, KI, ZQ, SA, MA-R, AA, SA-S, and FA-D. All authors contributed to the article and approved the submitted version.

## Conflict of Interest

The authors declare that the research was conducted in the absence of any commercial or financial relationships that could be construed as a potential conflict of interest.

## Publisher’s Note

All claims expressed in this article are solely those of the authors and do not necessarily represent those of their affiliated organizations, or those of the publisher, the editors and the reviewers. Any product that may be evaluated in this article, or claim that may be made by its manufacturer, is not guaranteed or endorsed by the publisher.
