# Supplementary material for: TERT Promoter Mutations Are an Independent Predictor of Distant Metastasis in Middle Eastern Papillary Thyroid Microcarcinoma
Source: Front Endocrinol (Lausanne). 2022 Mar 11;13:808298. doi: 10.3389/fendo.2022.808298 (PMC8962954; doi:10.3389/fendo.2022.808298)
Supplement: Supplementary file 1 [file Table_1.docx]

**Supplementary Table 1:** Primers used for *TERT* and *MAPK* genes (*HRAS*, *KRAS*, *NRAS* & *BRAF*) mutation analysis

| **Gene** | **Exon/region** | **Primer Sequence** |
| --- | --- | --- |
| *TERT* | Promoter | Forward: 5’-AGTGGATTCGCGGGCACAGA-3’ |
|  |  | Reverse: 5’-CAGCGCTGCCTGAAACTC-3’ |
| *HRAS* | Exon 1 | Forward: 5’-CAGGAGACCCTGTAGGAGGA -3’ |
|  |  | Reverse: 5’-CCCTATCCTGGCTGTGTCC -3’ |
|  | Exon 2 | Forward: 5’- GATTCCTACCGGAAGCAGGT -3’ |
|  |  | Reverse: 5’- TCACGGGGTTCACCTGTACT -3’ |
| *KRAS* | Exon 1 | Forward: 5’- AAGGCCTGCTGAAAATGACT-3’ |
|  |  | Reverse: 5’-TCAAAGAATGGTCCTGCACCAG-3’ |
|  | Exon 2 | Forward: 5’-TGTTTCTCCCTTCTCAGGATTC-3’ |
|  |  | Reverse: 5’-TTTAAACCCACCTATAATGGTGAA-3’ |
| *NRAS* | Exon 1 | Forward: 5’-GATGTGGCTCGCCAATTAAC-3’ |
|  |  | Reverse: 5’-CCGACAAGTGAGAGACAGGA-3’ |
|  | Exon 2 | Forward: 5’-CACCCCCAGGATTCTTACAG-3’ |
|  |  | Reverse: 5’-CACAAAGATCATCCTTTCAGAGAA-3’ |
| *BRAF* | Exon 15 | Forward: 5’- AAACTCTTCATAATGCTTGCTCTG -3’ |
|  |  | Reverse: 5’- TTTCTAGTAACTCAGCAGCATCTCA -3’ |

**Supplementary Table 2.** Clinico-pathological associations of *BRAF* mutation in papillary thyroid microcarcinoma

|  | **Total** | | ***BRAF* mutation** | | | | **p value** |
| --- | --- | --- | --- | --- | --- | --- | --- |
|  |  |  | **Present** | | **Absent** | |  |
|  | No. | % | No. | % | No. | % |  |
| **Total** | 184 |  | 84 | 45.7 | 100 | 54.3 |  |
| **Age at surgery (years)** |  |  |  |  |  |  |  |
| < 55 | 154 | 83.7 | 68 | 80.9 | 86 | 86.0 | 0.3568 |
| ≥ 55 | 30 | 16.3 | 16 | 19.1 | 14 | 14.0 |  |
| **Gender** |  |  |  |  |  |  |  |
| Male | 37 | 20.1 | 21 | 25.0 | 16 | 16.0 | 0.1298 |
| Female | 147 | 79.9 | 63 | 75.0 | 84 | 84.0 |  |
| **Histologic subtype** |  |  |  |  |  |  |  |
| Classical variant | 130 | 70.6 | 63 | 75.0 | 67 | 67.0 | 0.0140 |
| Follicular variant | 31 | 16.9 | 9 | 10.7 | 22 | 22.0 |  |
| Tall cell variant | 16 | 8.7 | 11 | 13.1 | 5 | 5.0 |  |
| Other variants | 7 | 3.8 | 1 | 1.2 | 6 | 6.0 |  |
| **Extrathyroidal extension** |  |  |  |  |  |  |  |
| Present | 63 | 34.2 | 39 | 46.4 | 24 | 24.0 | 0.0014 |
| Absent | 121 | 65.8 | 45 | 53.6 | 76 | 76.0 |  |
| **Lymphovascular invasion** |  |  |  |  |  |  |  |
| Present | 26 | 14.1 | 15 | 17.9 | 11 | 11.0 | 0.1842 |
| Absent | 158 | 85.9 | 69 | 82.1 | 89 | 89.0 |  |
| **Tumor focality** |  |  |  |  |  |  |  |
| Unifocal | 111 | 60.3 | 39 | 46.4 | 72 | 72.0 | 0.0004 |
| Multifocal | 73 | 39.7 | 45 | 53.6 | 28 | 28.0 |  |
| **Tumor laterality** |  |  |  |  |  |  |  |
| Unilateral | 121 | 65.8 | 46 | 54.8 | 75 | 75.0 | 0.0039 |
| Bilateral | 63 | 34.2 | 38 | 45.2 | 25 | 25.0 |  |
| **Lymph node metastasis** |  |  |  |  |  |  |  |
| Present | 85 | 51.8 | 48 | 63.2 | 37 | 42.1 | 0.0067 |
| Absent | 79 | 48.2 | 28 | 36.8 | 51 | 57.9 |  |
| **Distant metastasis** |  |  |  |  |  |  |  |
| Present | 11 | 6.0 | 3 | 3.6 | 8 | 8.0 | 0.1969 |
| Absent | 173 | 94.0 | 81 | 96.4 | 92 | 92.0 |  |
| **TNM Stage** |  |  |  |  |  |  |  |
| I | 165 | 90.7 | 73 | 86.9 | 92 | 93.9 | 0.3932 |
| II | 11 | 6.0 | 7 | 8.3 | 4 | 4.1 |  |
| III | 2 | 1.1 | 1 | 1.2 | 1 | 1.0 |  |
| IV | 4 | 2.2 | 3 | 3.6 | 1 | 1.0 |  |
| ***TERT* mutation** |  |  |  |  |  |  |  |
| Present | 16 | 8.7 | 10 | 11.9 | 6 | 6.0 | 0.1569 |
| Absent | 168 | 91.3 | 74 | 88.1 | 94 | 94.0 |  |
| **Recurrence** |  |  |  |  |  |  |  |
| Yes | 29 | 15.8 | 15 | 17.9 | 14 | 14.0 | 0.4753 |
| No | 155 | 84.2 | 69 | 82.1 | 86 | 86.0 |  |
